# Lower Urinary Tract Symptoms in Female Nurses: Evidence from the Nurse Urinary Related Health Study of China

**DOI:** 10.1155/2023/9207120

**Published:** 2023-04-30

**Authors:** Jie-Qiong Ren, Ming Li, Dong-Juan Xu, Jie Gao, Jun-Tao Chi, Min Yuan, Xing-Feng Lin, Hong-Xia Du, Chen Wu, Ke-Fang Wang

**Affiliations:** ^1^School of Nursing and Rehabilitation, Shandong University, Jinan, Shandong, China; ^2^School of Nursing, Purdue University, West Lafayette, IN, USA; ^3^Department of Nursing, Yantai Yuhuangding Hospital, Yantai, Shandong, China; ^4^Department of Nursing, Weifang People's Hospital, Weifang, Shandong, China; ^5^Department of Nursing, Second Hospital of Shandong University, Jinan, Shandong, China; ^6^Department of Nursing, Central Hospital Affiliated to Shandong First Medical University, Jinan, Shandong, China

## Abstract

**Aims:**

To estimate the prevalence and bother of lower urinary tract symptoms (LUTS) and the work-related and individual factors associated with LUTS among a representative sample of female nurses.

**Background:**

A healthy nursing workforce is essential to advance global health goals, especially during times of extraordinary demand for nursing care. LUTS frequently occur and persist in women and are correlated with multiple negative health outcomes and diminished work engagement and productivity. However, the study of LUTS among female nurses failed to receive sufficient attention from researchers.

**Methods:**

We used baseline data for 13,191 female nurses in China collected for the prospective cohort study, the Nurse Urinary Related Health Study (NURS). We assessed nurses' self-reported LUTS and symptom-related bother using the International Consultation on Incontinence Questionnaire-Female LUTS. We used descriptive statistics to summarize LUTS prevalence and its related bother and a mixed-effects logistic regression model to test the effects of work-related and individual factors on LUTS.

**Results:**

Most of the participants in this study were younger than 40 years old (82.9%), were married (74.8%), and had given birth once or never (73.7%). Few participants had chronic diseases (3.4%), consumed alcohol (7.3%), smoked (0.4%), or had overweight/obesity (27.7%). The prevalence of any LUTS was 51.1%, and over 50% of the nurses with LUTS in this study had experienced moderate or severe bother, except for urinary frequency. Working longer than five years, more than 40 hours per week, and in Level A, major tertiary hospitals were found to be risk factors of LUTS, and a nurse-to-bed ratio higher than 0.40 was found to be a protective factor. Increased fluid intake also was found to be a protective factor of LUTS in nurses, and having chronic constipation was found to be a risk factor.

**Conclusions:**

LUTS are highly prevalent and severely bothersome among female nurses in China, despite the fact that the female nurses in this study were relatively young, healthy, had few childbirths, and were living healthy lifestyles. This finding warrants remedial action that is related to both behavioral and environmental factors to ensure a healthy nursing workforce. Nurses cannot reasonably be expected to contribute to global health when one of their basic needs as humans, i.e., the normal function of urinary elimination, is ignored. *Implications for Nursing Management*. Nurse managers should be aware that nurses' basic needs regarding urination are negatively affected by LUTS and related bother. Nurse managers should address LUTS-related problems on multiple levels, including overseeing and reporting LUTS in nurses, exploring innovative care models to mitigate the negative effects of excessive workloads on nurses, and remodeling the nursing culture and encouraging positive coping behaviors for the self-health of nurses.

## 1. Background

Urinary elimination is a basic human function that can be compromised by lower urinary tract symptoms (LUTS), which include nocturia, urinary urgency, bladder pain, urinary frequency, hesitancy, straining, intermittency, and urinary incontinence [1]. LUTS are very prevalent health conditions that especially affect the female population. Global estimations indicate that approximately 1.6 billion (46.8%) women have at least one type of LUTS [2]. Symptom experience, i.e., LUTS “bother,” has been found to be associated with multiple negative health outcomes, including distress, shame, social isolation [3], and diminished sexual health, emotional well-being, and quality of life [4–6]. In addition, LUTS are associated with heavy economic burden due to the costs of professional therapy and routine care [7]. LUTS are therefore a pressing health concern in the female population.

Female nurses are the focus population for this LUTS study. Female nurses are susceptible to LUTS, as evidenced by the prevalence rate of LUTS in small samples of nurses that ranges from 65% to 90% [8, 9], which is a higher prevalence rate than in the general female population. Nurses play a vital role in global health promotion [10, 11], which is already challenged by the worldwide shortage of nurses. Having nurses with LUTS and who experience symptom-related bother might exacerbate this situation, as LUTS and bother of LUTS have been found to be significantly associated with reduced work engagement and productivity in female workers [12].

The study of LUTS among female nurses has failed to receive sufficient attention from researchers. Only a few LUTS, in particular urinary incontinence and urinary urgency, have been investigated among representative nurse samples, and these studies were limited to developed countries such as the United States and Japan [13, 14]. The characteristics of nurses working in developed countries differ from those of nurses working in developing countries. For example, frontline nurses in China typically are younger than nurses working in developed countries [13–15]. LUTS are associated with age, and thus, prevalence findings from developed countries may not be able to be extrapolated to nurses working in developing countries. In addition, the literature indicates that little is known about LUTS bother in female nurses. Despite the fact that the health of nurses is often negatively influenced by hazardous and risk-related working conditions, the evidence for LUTS is limited primarily to intrinsic determinants of LUTS, such as advancing age, marital status, and parity [16]. Thus, the effects of work-related factors on LUTS in representative samples of female nurses remain unclear.

To address these research gaps and accumulate evidence that will be beneficial for mitigating LUTS in nurses, we analyzed baseline data collected in an ongoing prospective cohort study of a representative sample of female nurses in China, i.e., the Nurse Urinary Related Health Study (NURS), to undertake the following three research objectives: (1) describe the prevalence of LUTS in a representative sample of female nurses, (2) document the perceived bother of female nurses with LUTS, and (3) quantify the correlation of work-related factors with LUTS among female nurses.

## 2. Methods

### 2.1. Design and Participants

**Figure 1:**
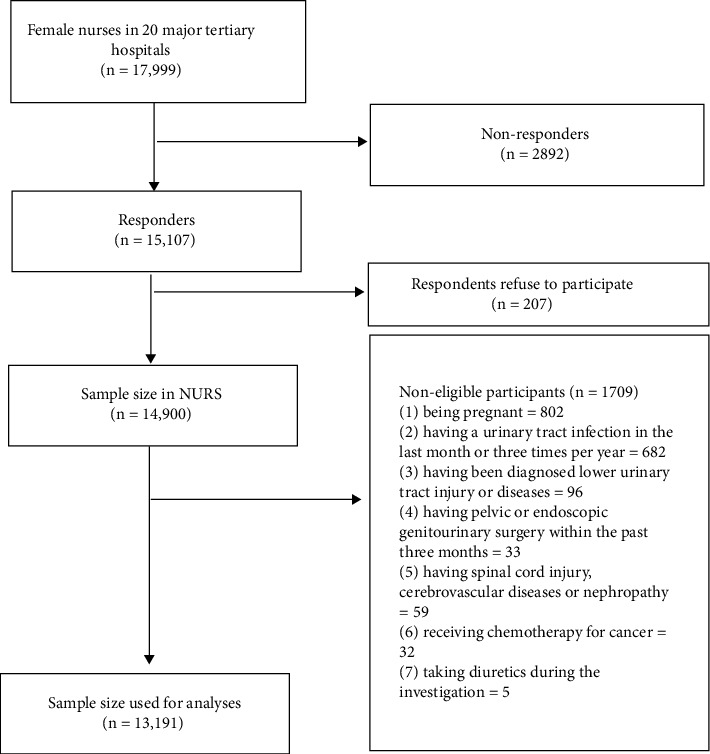
Study flowchart.

The inaugural survey of NURS was conducted between November 2020 and February 2021 in Shandong Province, China. A two-stage cluster sampling design was used to recruit eligible nurses for NURS. The 17 prefecture-level administrative regions in Shandong Province were stratified into four categories, i.e., high, medium-high, medium-low, and low developed regions, based on the quartile spacing of their gross domestic product in 2019. Tertiary hospitals in China are categorized into three subsidiary levels, A, B, and C, based primarily on the number of hospital beds, level of quality of care, and available medical technologies. Most nurses in China are employed in Level A and Level B major tertiary hospitals, and therefore, the number of level A and level B major tertiary hospitals per region was collected to create a contingency table (Table 1), and 20% of hospitals in each cell were randomly sampled using a random number generator. As depicted in Figure 1, a total of 20 hospitals were sampled and contact was made with all female registered nurses (*n* = 17,999) from these hospitals unless they failed to respond (*n* = 2892) or refused to participate (*n* = 207). The exclusion criteria for this study were set as follows: (1) being pregnant, (2) having a urinary tract infection in the last month or three times within the past year, (3) having been diagnosed with a lower urinary tract injury or disease, (4) having pelvic or endoscopic genitourinary surgery within the past three months, (5) receiving chemotherapy for cancer, (6) having a spinal cord injury, cerebrovascular disease, or nephropathy, and (7) taking diuretics during the investigation. The final sample for this analysis was 13,191 female nurses.

All parties involved respected the relevant principles stipulated in the Declaration of Helsinki [17]. The ethical oversight of NURS was obtained from the institutional review boards of the researchers' university (No. 2019-R-021). A detailed description of NURS can be found in the following weblink: https://www.nursing.sdu.edu.cn/info/1170/3676.htm.

### 2.2. Data Collection

NURS uses the e-survey to collect data. To facilitate data collection, the principal investigator contacted the director of the nursing department in each of the 20 hospitals and obtained hospital-level informed consent. The director of the nursing department provided a spreadsheet with the total number of full-time equivalent registered nurses at the hospital for the investigator's reference. Each director was asked to recommend at least two coordinators to facilitate access to the e-survey for all nurses. The e-survey was created on WJX.cn, an online survey tool. The first screen of the e-survey presented information about the study. Then, the participant was asked to check a tick-box to give informed consent before proceeding. The expiration of the web link was set at two weeks from the initial opening, and no incentives for respondents were provided.

### 2.3. Measures

#### 2.3.1. LUTS and Symptom-Related Bother

The International Consultation on Incontinence Questionnaire-Female LUTS was used with permission obtained from the Bristol Urological Institute, UK to assess nurses' LUTS and symptom-related bother [18]. Eleven items were used to assess symptoms of nocturia, urgency, bladder pain, daytime frequency, hesitancy, straining, intermittency, urgency urinary incontinence, stress urinary incontinence, unexplained urinary incontinence, and nocturnal enuresis experienced by individuals during the past four weeks [19]. Of these 11 symptoms, nocturia was defined as at least two times of urination per night (not during the night shift) [20]; frequency was defined as urinating more than eight times during the day (not during the night shift) [1]; and all other symptoms were graded on a five-point scale of never, occasionally, sometimes, most of the time, and all of the time. Nurses with responses of never and occasionally were categorized as having no such symptom [20]. If nurses reported at least one type of LUTS, they would be coded as having LUTS.

Along with each symptom, an additional item was designed for the International Consultation on Incontinence Questionnaire-Female LUTS to capture individuals' perceived symptom-specific bother. Bother was graded on a visual analogue scale that ranged from 0 (not at all) to 10 (a great deal). Nurses' responses were categorized into no bother (score of 0), minor bother (score of 1–4), moderate bother (score of 5–7), and severe bother (score of 8–10) [21].

#### 2.3.2. Work-Related Characteristics

Multiple self-reported work-related characteristics were assessed and included years of work (≤5, 5–10, >10), average work hours per week (≤40, >40), night shift in the past six months (yes, no), professional title (senior nurse or below, supervisor nurse or above), hospital-level (Level B major tertiary hospital, Level A major tertiary hospital), and department type (internal medicine, surgery, operating room, intensive care unit, out-patient and emergency, pediatrics, obstetrics and gynecology, nursing administration, and others). In addition, the principal investigator calculated the nurse-to-bed ratio variable using the number of full-time equivalent registered nurses divided by the number of beds in a hospital. The National Care Nursing Development Plan of China recommends that the nurse-to-bed ratio of major tertiary hospitals should shift from 0.60 to 0.80, but very few of the major tertiary hospitals across the country have been able to meet this standard. Therefore, we categorized the nurse-to-bed ratio into three levels based on the minimum ratio of 0.40 and the formerly recommended ratio of 0.60. As such, the nurse-to-bed ratio was categorized into <0.40, 0.40 to 0.60, and ≥0.60.

#### 2.3.3. Individual Characteristics

Data were collected for self-reported individual characteristics, including demographic characteristics and health-related characteristics. The demographic characteristics assessed include age, race/ethnicity (Han Chinese, others), educational background (below bachelor's degree, bachelor's degree or above), marital status (single/separated/divorced/widowed, married), and monthly income (≤RMB 6000, >RMB 6000). Data also were collected for health-related characteristics that are known to be associated with LUTS [22]: parity (0, 1, ≥2), average fluid intake per day (≤500 ml, 500 ml–1500 ml, >1500 ml), body mass index (BMI) (underweight/normal, overweight/obesity), chronic constipation (yes/no), alcohol use (yes/no), smoking (yes/no), taking part in physical exercise (yes/no), and chronic disease (yes/no). Data were collected for four chronic diseases that are associated with LUTS: hypertension, heart disease, diabetes, and hyperlipidemia [16]. Nurses with any of the four chronic diseases were coded as “yes” and those without any of these conditions were coded as “no.”

### 2.4. Statistical Analysis

The characteristics of nurses are described in terms of mean ± standard deviation or frequencies and percentages, as appropriate. The prevalence of LUTS and nurses' perceived symptom-specific bother are presented as numbers and percentages with 95% confidence interval (95% CI). A mixed-effects logistic regression model was used to regress individual and work-related characteristics (fixed effects), hospital ID (i.e., 1 to 20), and department type (random effects) on LUTS. Cases with missing values were excluded in the model. All analyses were performed using Stata SE 16.0, and *p*  <  0.05 indicates statistical significance.

## 3. Results

### 3.1. Participants

Table2presents a summary of the detailed characteristics of the sample. The mean age of the 13,191 female nurses at the time of the survey was 32.67 ± 7.52 years (range: 19–60 years), with the majority younger than 40 years old (82.9%, 95% CI = 82.3–83.6). Most participants were married (74.8%) and had given birth once or never (73.7%). Very few participants had chronic diseases (3.4%), consumed alcohol (7.3%), or smoked (0.4%). Fewer than 30% of the female nurses in the sample had overweight/obesity. More than 70% had a fluid intake of less than or equal to 1500 ml fluid per day, and more than 26% had chronic constipation. The nurse-to-bed ratio ranged from 0.36 to 0.79, and more than 30% of the female nurses in the sample worked in hospitals with a nurse-to-bed ratio below 0.60. Approximately 50% of the nurses reported that they work more than 40 hours per week, and the mean work hours per week of female nurses was approximately 45 hours. More than 60% of nurses worked in a Level A major tertiary hospital, and roughly 28% of female nurses had worked in hospitals for less than five years.

### 3.2. Prevalence of LUTS

Table 3 presents a summary of the prevalence and symptom-specific bother of LUTS reported by the female nurses in this study. The prevalence of LUTS is 51.1% (95% CI = 50.2–51.9). Of all LUTS, urinary urgency is the most prevalent symptom (26.1%, 95% CI = 25.3–26.8), followed by stress urinary incontinence (17.9%, 95% CI = 17.2–18.5), nocturia (13.9%, 95% CI = 13.3–14.5), urgency urinary incontinence (12.2%, 95% CI = 11.6–12.7), and hesitancy (11.9%, 95% CI = 11.3–12.4). Frequency is the least prevalent symptom of LUTS found for female nurses (0.7%, 95% CI = 0.5–0.8).

### 3.3. Symptom-Specific Bother of LUTS

Table 3 also presents the prevalence of LUTS bother. Except for frequency, more than 90% of nurses with LUTS reported at least minor bother, and more than 50% of nurses with LUTS reported moderate bother and severe bother. Nurses with unexplained urinary incontinence were most likely to report at least minor bother (97.9%) and at least moderate bother (77.1%). Other LUTS that were likely to be reported by nurses as at least moderate bother include nocturnal enuresis (71.6%), urgency urinary incontinence (70.3%), bladder pain (69.5%), stress urinary incontinence (69.5%), and straining (63.9%). Stress urinary incontinence was most likely to be reported as severe bother by nurses (36.9%), followed by unexplained urinary incontinence (33.3%) and urgency urinary incontinence (32.3%).

### 3.4. Work-Related Factors Associated with LUTS

Table 4 presents a summary of the work-related factors associated with LUTS. Nurses who had worked five to ten years (OR = 1.244, 95% CI = 1.112–1.391) or longer than ten years (OR = 1.246, 95% CI = 1.076–1.444) and worked more than 40 hours per week (OR = 1.264, 95% CI = 1.175–1.360) were likely to have LUTS. Nurses who were working in a Level A major tertiary hospital were more likely to have LUTS than those working in a Level B major tertiary hospital (OR = 1.226, 95% CI = 1.111–1.352). Compared with nurses who worked in a hospital with a nurse-to-bed ratio less than 0.40, those working in a hospital with a nurse-to-bed ratio of 0.40 to 0.60 (OR = 0.626, 95% CI = 0.503–0.779) and 0.60 and above (OR = 0.647, 95% CI = 0.518–0.808) were less likely to have LUTS.

### 3.5. Individual Factors Associated with LUTS

Table 4 also presents individual factors associated with LUTS. Female nurses who were aged 40 years and older (OR = 1.147, 95% CI = 1.005–1.309), married (OR = 1.161, 95% CI = 1.015–1.329), and had given birth two or more times (OR = 1.609, 95% CI = 1.388–1.866) are associated with having LUTS. Overweight and obese (OR = 1.208, 95% CI = 1.114–1.311), having chronic disease (OR = 1.564, 95% CI = 1.267–1.930), and alcohol use (OR = 1.258, 95% CI = 1.095–1.444) increase the odds of nurses having LUTS whereas taking part in physical exercise (OR = 0.819, 95% CI = 0.745–0.901) reduces the odds of nurses having LUTS. Compared with nurses with an average fluid intake of less than 500 ml per day, those who consumed 500 ml to 1500 ml per day (OR = 0.803, 95% CI = 0.726–0.888) and more than 1500 ml per day (OR = 0.732, 95% CI 0.652–0.822) were less likely to have LUTS. Having chronic constipation (OR = 1.812, 95% CI = 1.670–1.967) increased the odds of nurses having LUTS.

## 4. Discussion

The inaugural survey of NURS in China was launched successfully and has provided important findings about the prevalence, bother, and risk factors of LUTS in a representative sample of female nurses. We found the prevalence rate of LUTS in female nurses to be 51.1%, and more than half of these nurses perceived at least moderate bother from all LUTS except urinary frequency. We identified four work-related risk factors of LUTS in nurses: working more than 40 hours per week, working longer than five years, working in Level A major tertiary hospitals, and working in hospitals with inadequate staffing (bed-to-nurse ratio less than 0.4). In addition, we found that an increase in fluid intake is a protective factor for LUTS in nurses and having chronic constipation is a risk factor for LUTS.

Although the female nurses in this study were relatively young, healthy, had few childbirths, and lived healthy lifestyles, the overall LUTS prevalence rate of 51.1% for this group is much higher than the prevalence rate found for women in general in both developing countries [20] and developed countries [23]. Our findings validate the conclusion found in small sample studies [8, 9, 24]. In addition, we found that more than 10% of nurses experienced urinary urgency, stress urinary incontinence, nocturia, urgency urinary incontinence, and hesitancy, and the prevalence of urinary urgency (26.1%) and stress urinary incontinence (17.9%) is greater than that found in the general female population both in China [20] and in developed countries [23]. Collectively, the findings from this study corroborate the need for female nurses to be a focus population for LUTS studies.

Symptom perception is an important variable, as it might influence individuals' health-related outcomes [25]. In NURS, the symptom perception of LUTS, i.e., bother, was reported by a large proportion of nurses. Specifically, we found that almost all the nurses in the study had bother perception, and 50% of nurses even perceived moderate bother and more severe bother from having LUTS, excluding urinary frequency. Moreover, more than 30% of the nurses with urinary incontinence reported severe symptom bother. These findings differ from those for women in general who often perceive no bother or minor bother from having LUTS [21]. This discrepancy between female nurses and the general female population might be attributable to nurses' training and work experiences. Nurses are trained at school and in clinical settings to be sensitive to symptoms of various diseases, which might enhance their perceptions of LUTS. Also, LUTS that interfere with nurses' concentration on their work might also be perceived as troublesome to nurses. Collectively, these findings indicate that female nurses bear substantial burden and face significant challenges related to LUTS. To develop a robust nursing workforce, LUTS should be addressed, and in-depth studies of bother may inform the better management of LUTS in nurses.

We found direct evidence that supports significant associations between work-related factors and LUTS in nurses. Heavy workloads of hospital nurses are challenges in many healthcare systems. In this study, we found that the nurse-to-bed ratio ranged from 0.36 to 0.79, which is far below that of most countries [26]. Moreover, we found that 4.0% of nurses in China are working in hospitals with a nurse-to-bed ratio that is under the national minimum ratio of 0.40. Despite years of effort from the government [27], the nurse shortage in China remains more severe compared to that in developed countries. In addition, we found that almost 50% of nurses work overtime, i.e., work more than 40 hours per week, and the mean work hours per week of female nurses is approximately 45 hours, which is much more than the mean work hours of nurses in developed countries such as Germany [28] and the United States [29]. Overtime work is a well-known phenomenon in the academic world [30, 31], but it has not been formally documented in clinical practice, which limits the government's protective measures for employees to function. Both low nurse-to-bed ratios and overtime work are problems that are worthy of attention, as we found that these factors are significantly associated with LUTS in female nurses. In addition, to facilitate the implementation of reporting staffing ratios through a public reporting system, the government should work with medical facilities to develop other strategies to ensure an adequate minimum staffing ratio standard and promote the achievement of the recommended ratio. As for overtime work, the government should require employers to negotiate their work hours and overtime conditions for specific positions and to write them clearly into the labor contract. Local changes, e.g., changing the model of care in individual nursing units, also should be explored.

We also found that working in a Level A major tertiary hospital and working for a long duration (in years) are risk factors for LUTS in nurses. Nurses who work in high-level hospitals in China were reported to be exposed continuously to a large number of hospital visits by patients, high hospitalization rates, more hospitalized patients, and patients with complex health conditions [32]. A long duration of working may also represent the cumulative experiences of nurses in handling patients with various conditions and being assigned complex patients and/or roles, which can lead to stress. Such exposure to stress may result in chronic stress, which has been found to be a validated risk factor for LUTS [33]. Future studies may want to corroborate these findings and test the role of stress on relationships between work-related risk factors and LUTS in nurses.

Multiple intrinsic determinants of LUTS that are found in the general population of women were validated in our nurse sample. Although nurses are knowledgeable with regard to healthy behaviors and health improvement strategies [34], we found that two risk factors, inadequate fluid intake and constipation, are more severe in our sample than in the general population of women [35, 36]. The inconsistency between nurses' knowledge and their behavior indicates that occupational hazards exist in nursing work and nurses' work environments. Nurses are trained to prioritize patients' needs over their own, and when they are busy, they may ignore their personal needs to drink water and go to the restroom. The culture of caring for others at the expense of self needs to shift to a better balance. In a risk-associated working environment, strategies should be developed to empower nurses not only to meet the health needs of the population, but also to take care of their own health.

This study has two primary strengths. First, with the study's representative female nurse sample and high response rate, we were able to examine the LUTS profile of nurses who were working in lower- and middle-income countries, which enriches the evidence of LUTS in nurses in global scenarios. Second, this study adds to the literature by finding multiple modifiable work-related factors that are relevant to LUTS in nurses, which would complement existing interventions to relieve the burden of LUTS for nurses.

This study has several limitations. The first limitation is the inherent limitation of a cross-sectional study in which we could not determine the nature of relationships between work-related factors and LUTS. Using data that will be collected in the NURS follow-up visits, we will be able to look at the incidence and trajectories of LUTS, and such information will help reveal these relationships. Second, the representative sample is within one province of China, Shandong. Shandong Province has the nation's second largest number of tertiary hospitals, and the number of registered nurses in Shandong is approximately 0.3765 million, representing almost 8.5% of all nurses (4.45 million) across the nation [15]. The sample characteristics are comparable to those reported in the China Health Statistics Yearbook 2020 [15]. Last, the NURS data collection was initiated during the COVID-19 pandemic and, despite the control of the pandemic in China, the mandatory mask policy and visiting restriction policy were maintained in hospitals. The mandatory mask policy may have influenced the need for hydration in nurses and the visitation policy may have increased the workload of nurses. These policies may introduce noise to our findings.

## 5. Conclusions

A healthy nursing workforce is an essential building block for robust healthcare systems and is the cornerstone for advancing global health. LUTS are prevalent and bothersome for female nurses and create challenges for a healthy nursing workforce to deliver high-quality care. We cannot ask nurses to contribute even more to global health when one of their basic needs as humans, i.e., the normal function of urinary elimination, is ignored. More studies are needed to contribute compelling evidence to reveal the impacts of modifiable individual and work-related factors on nurses' LUTS and associated bother. All such efforts will eventually lead to interventions for mitigating LUTS in nurses.

## 6. Implications for Nursing Management

The findings from this study have several clinical implications. Nurse managers should be aware that nurses' basic needs as humans are compromised due to LUTS and related bother, and addressing LUTS-related problems requires significant effort on multiple fronts. As the representatives of thousands of nurses, nurse managers should oversee the bladder health of nurses and report the problem of LUTS to stakeholders when necessary. Such efforts may directly mitigate LUTS in nurses. Nurse managers also should explore innovative care models within departments or hospitals to increase productivity and alleviate the negative effects of excessive workloads on nurses as an indirect pathway to mitigate LUTS. In addition, nurse managers may also employ strategies to remodel the nursing culture and encourage positive coping behaviors in nurses. In doing so, clinical nurses will be able to take care of themselves while also providing quality care to patients.

## Figures and Tables

**Table 1:** The number of level A and level B major tertiary hospitals across regions with diverse GDP.

Regions	Regions with high GDP	Regions with medium-high GDP	Regions with medium-low GDP	Regions with low GDP	Total number
Hospital level					
Level A major tertiary hospital	20	10	9	9	48
Level B major tertiary hospital	19	10	8	11	48

**Table 2:** Description of participants' characteristics.

Variables	Total (*n* = 13,191)		With LUTS (*n* = 6735)		Without LUTS (*n* = 6456)	
	Mean ± SD or *n* (%)	95% CI	Mean ± SD or *n* (%)	95% CI	Mean ± SD or *n* (%)	95% CI
Age (years)	32.67 ± 7.52	32.54–32.80	33.59 ± 7.53	33.41–33.77	31.72 ± 7.38	31.54–31.90
Age						
<40 years	10,940 (82.9)	82.3–83.6	5443 (80.8)	79.9–81.7	5497 (85.1)	84.3–86.0
≥40 years	2251 (17.1)	16.4–17.7	1292 (19.2)	18.3–20.1	959 (14.9)	14.0–15.7
Race/ethnicity						
Han Chinese	13,068 (99.1)	98.9–99.2	6663 (98.9)	98.7–99.2	6405 (99.2)	99.0–99.4
Other	123 (0.9)	0.8–1.1	72 (1.1)	0.8–1.3	51 (0.8)	0.6–1.0
Educational background						
Below bachelor degree	2662 (20.2)	19.5–20.9	1229 (18.2)	17.3–19.2	1433 (22.2)	21.2–23.2
Bachelor degree or higher	10,529 (79.8)	79.1–80.5	5506 (81.8)	80.8–82.7	5023 (77.8)	76.8–78.8
Marital status						
Single, separated, divorced, or widowed	3319 (25.2)	24.4–25.9	1275 (18.9)	18.0–19.9	2044 (31.7)	30.5–32.8
Married	9872 (74.8)	74.1–75.6	5460 (81.1)	80.1–82.0	4412 (68.3)	67.2–69.5
Monthly income						
≤RMB 6000	7701 (58.4)	57.5–59.2	3752 (55.7)	54.5–56.9	3949 (61.2)	60.0–62.4
>RMB 6000	5490 (41.6)	40.8–42.5	2983 (44.3)	43.1–45.5	2507 (38.8)	37.6–40.0
Parity^a^						
0	4110 (31.5)	30.7–32.3	1637 (24.4)	23.4–25.5	2473 (38.9)	37.7–40.1
1	5515 (42.2)	41.4–43.1	3049 (45.5)	44.3–46.7	2466 (38.8)	37.6–40.0
≥2	3434 (26.3)	25.5–27.1	2014 (30.1)	29.0–31.2	1420 (22.3)	21.3–23.4
BMI^a^						
Underweight/normal	9524 (72.3)	71.5–73.0	4669 (69.4)	68.3–70.5	4885 (75.3)	74.2–76.3
Overweight/obesity	3654 (27.7)	27.0–28.5	2059 (30.6)	29.5–31.7	1595 (24.7)	23.7–25.8
Chronic diseases						
No	12,747 (96.6)	96.3–96.9	6442 (95.6)	95.1–96.1	6305 (97.7)	97.3–98.0
Yes	444 (3.4)	3.1–3.7	293 (4.4)	3.9–4.9	151 (2.3)	2.0–2.7
Chronic constipation						
No	9675 (73.3)	72.6–74.1	4527 (67.2)	66.1–68.3	5148 (79.7)	78.7–80.7
Yes	3516 (26.7)	25.9–27.4	2208 (32.8)	31.7–33.9	1308 (20.3)	19.3–21.3
Alcohol use						
No	12,228 (92.7)	92.2–93.1	6193 (92.0)	91.3–92.6	6035 (93.5)	92.9–94.1
Yes	963 (7.3)	6.9–7.8	542 (8.0)	7.4–8.7	421 (6.5)	5.9–7.1
Smoking						
No	13,145 (99.6)	99.5–99.7	6711 (99.6)	99.5–99.8	6434 (99.7)	99.5–99.8
Yes	46(0.4)	0.3–0.5	24(0.4)	0.2–0.5	22 (0.3)	0.2–0.5
Average fluid intake per day^c^						
≤500 ml	2160 (16.4)	15.8–17.0	1202 (17.8)	17.0–18.8	958 (14.8)	14.0–15.7
500 ml–1500 ml	7563 (57.3)	56.5–58.2	3816 (56.7)	55.5–57.8	3747 (58.0)	56.8–59.2
>1500 ml	3468 (26.3)	25.5–27.0	1717 (25.5)	24.5–26.5	1751 (27.1)	26.1–28.2
Taking part in physical exercise						
No	10,695 (81.1)	80.4–81.7	5563 (82.6)	81.7–83.5	5132 (79.5)	78.5–80.5
Yes	2496 (18.9)	18.3–19.6	1172 (17.4)	16.5–18.3	1324 (20.5)	19.5–21.5
Working years						
≤5	3710 (28.1)	27.4–28.9	1476 (21.9)	20.9–22.9	2234 (34.6)	33.5–35.8
5–10	4620 (35.0)	34.2–35.8	2500 (37.1)	36.0–38.3	2120 (32.8)	31.7–34.0
>10	4861 (36.9)	36.0–37.7	2759 (41.0)	39.8–42.1	2102 (32.6)	31.4–33.7
Professional title^b^						
Senior nurse or below	8357 (63.4)	62.5–64.2	3993 (59.3)	58.1–60.5	4364 (67.6)	66.4–68.7
Supervisor nurse or higher	4834 (36.6)	35.8–37.5	2742 (40.7)	39.5–41.9	2092 (32.4)	31.3–33.6
Work hours per week (hours/week)	44.57 ± 8.00	44.43–44.70	45.00 ± 8.16	44.80–45.19	44.11 ± 7.81	43.93–44.31
Work hours per week						
≤40	7109 (53.9)	53.0–54.7	3433 (51.0)	49.8–52.2	3676 (56.9)	55.7–58.1
>40	6082 (46.1)	45.3–47.0	3302 (49.0)	47.8–50.2	2780 (43.1)	41.9–44.3
Night shift in the past 6 months						
No	4788 (36.3)	35.5–37.1	2530 (37.6)	36.4–38.7	2258 (35.0)	33.8–36.1
Yes	8403 (63.7)	62.9–64.5	4205 (62.4)	61.3–63.6	4198 (65.0)	63.9–66.2
Hospital level						
Level B major tertiary hospital	4885 (37.0)	36.2–37.9	2361 (35.1)	33.9–36.2	2524 (39.1)	37.9–40.3
Level A major tertiary hospital	8306 (63.0)	62.1–63.8	4374 (64.9)	63.8–66.1	3932 (60.9)	59.7–62.1
Nurse-to-bed ratio^c^						
0.36 to < 0.40	529 (4.0)	3.7–4.4	320 (4.8)	4.3–5.3	209 (3.2)	2.8–3.7
0.40–0.60	3822 (29.0)	28.2–29.8	1871 (27.8)	26.7–28.9	1951 (30.2)	29.1–31.4
≥0.60 to 0.79	8840 (67.0)	66.2–67.8	4544 (67.5)	66.3–68.6	4296 (66.5)	65.4–67.7
Department type^c^						
Internal medicine	4101 (31.1)	30.3–31.9	2125 (31.6)	30.5–32.7	1976 (30.6)	29.5–31.7
Surgery	2932 (22.2)	21.5–22.9	1492 (22.2)	21.2–23.2	1440 (22.3)	21.3–23.3
Operating room	709 (5.4)	5.0–5.8	375 (5.6)	5.0–6.1	334 (5.2)	4.7–5.7
Intensive care unit	877 (6.6)	6.2–7.1	426 (6.3)	5.8–6.9	451 (7.0)	6.4–7.6
Out-patient and emergency	1152 (8.7)	8.3–9.2	600 (8.9)	8.3–9.6	552 (8.6)	7.9–9.3
Pediatrics	891 (6.8)	6.3–7.2	442 (6.6)	6.0–7.2	449 (7.0)	6.4–7.6
Obstetrics and gynecology	1149 (8.7)	8.2–9.2	597 (8.9)	8.2–9.6	552 (8.6)	7.9–9.3
Nursing administration	446 (3.4)	3.1–3.7	231 (3.4)	3.0–3.9	215 (3.3)	2.9–3.8
Other	934 (7.1)	6.7–7.5	447 (6.6)	6.1–7.3	487 (7.5)	6.9–8.2

Note: LUTS: lower urinary tract symptoms. With LUTS: having at least one type of lower urinary tract symptoms. Without LUTS: not having any type of lower urinary tract symptoms. ^a^Variables with missing data: parity (*n* = 132) and BMI (*n* = 13). ^b^Professional title: There are five grades of registered nurses in Mainland China. Senior or below indicates grades 1 and 2 registered nurses, and supervisor nurses or above indicates grades 3, 4, and 5 registered nurses. ^c^Percentages are rounded to the nearest percent. 1 RMB = 0.1494 US dollars.

**Table 3:** Prevalence and symptom-specific bother of lower urinary tract symptoms.

Symptoms	LUTS (*n* = 13,191)		Symptom-specific bother of LUTS (*n* = 6735)							
			No		Minor		Moderate		Severe	
	*n* (%)	95% CI	*n* (%)	95% CI	*n* (%)	95% CI	*n* (%)	95% CI	*n* (%)	95% CI
Nocturia	1833 (13.9)	13.3–14.5	180 (9.8)	8.5–11.3	617 (33.7)	31.5–35.9	668 (36.4)	34.2–38.7	368 (20.1)	18.3–22.0
Urinary urgency	3437 (26.1)	25.3–26.8	271 (7.9)	7.0–8.8	1230 (35.8)	34.2–37.4	1366 (39.7)	38.1–41.4	570 (16.6)	15.4–17.9
Bladder pain	1056 (8.0)	7.5–8.5	40 (3.8)	2.7–5.1	282 (26.7)	24.1–29.5	485 (45.9)	42.9–49.0	249 (23.6)	21.0–26.3
Frequency	86 (0.7)	0.5–0.8	29 (33.7)	23.9–44.7	22 (25.6)	16.8–36.1	24 (27.9)	18.8–38.6	11 (12.8)	6.6–21.7
Hesitancy^a^	1563 (11.9)	11.3–12.4	140 (9.0)	7.6–10.5	576 (36.9)	34.5–39.3	643 (41.1)	38.7–43.6	204 (13.1)	11.4–14.8
Straining^a^	883 (6.7)	6.3–7.1	35 (4.0)	2.8–5.5	284 (32.2)	29.1–35.4	397 (45.0)	41.6–48.3	167 (18.9)	16.4–21.7
Intermittency^a^	1032 (7.8)	7.4–8.3	79 (7.7)	6.1–9.4	362 (35.1)	32.2–38.1	422 (40.9)	37.9–44.0	169 (16.4)	14.2–18.8
UUI^a^	1603 (12.2)	11.6–12.7	51 (3.2)	2.4–4.2	426 (26.6)	24.4–28.8	609 (38.0)	35.6–40.4	517 (32.3)	30.0–34.6
SUI	2356 (17.9)	17.2–18.5	68 (2.9)	2.2–3.6	651 (27.6)	25.8–29.5	767 (32.6)	30.7–34.5	870 (36.9)	35.0–38.9
Unexplained UI	571 (4.3)	4.0–4.7	12 (2.1)	1.1–3.6	119 (20.8)	17.6–24.4	250 (43.8)	39.7–48.0	190 (33.3)	29.4–37.3
Nocturnal enuresis	176 (1.3)	1.1–1.5	4 (2.3)	0.6–5.7	46 (26.1)	19.8–33.3	84 (47.7)	40.2–55.4	42 (23.9)	17.8–30.9
Any LUTS	6735 (51.1)	50.2–51.9								

Note: UUI: urgency urinary incontinence. SUI: stress urinary incontinence. Unexplained UI: unexplained urinary incontinence. Any LUTS: at least one type of lower urinary tract symptoms. ^a^Percentages of bother are rounded to the nearest percent.

**Table 4:** Factors associated with lower urinary tract symptoms in female nurses (*n* = 13,046).

Variables	OR	95% CI	*p*
Age (ref. = <40 years)			
≥40 years	1.147	1.005–1.309	0.042
Race/ethnicity (ref. = Han Chinese)			
Other	1.342	0.926–1.944	0.120
Educational background (ref. = below bachelor degree)			
Bachelor degree or higher	0.944	0.856–1.041	0.251
Marital status (ref. = single, separated, divorced, or widowed)			
Married	1.161	1.015–1.329	0.030
Monthly income (ref. = ≤RMB 6000.00)			
>RMB 6000.00	1.060	0.975–1.153	0.169
Parity (ref. = 0)			
1	1.466	1.283–1.676	<0.001
≥2	1.609	1.388–1.866	<0.001
BMI (ref. = underweight/normal)			
Overweight/obesity	1.208	1.114–1.311	<0.001
Chronic disease (ref. = no)			
Yes	1.564	1.267–1.930	<0.001
Chronic constipation (ref. = no)			
Yes	1.812	1.670–1.967	<0.001
Alcohol use (ref. = no)			
Yes	1.258	1.095–1.444	0.001
Smoking (ref. = no)			
Yes	1.168	0.640–2.132	0.612
Taking part in physical exercise (ref. = no)			
Yes	0.819	0.745–0.901	<0.001
Average fluid intake per day (ref. = ≤500 ml)			
500 ml–1500 ml	0.803	0.726–0.888	<0.001
>1500 ml	0.732	0.652–0.822	<0.001
Working years (ref. = ≤5)			
5–10	1.244	1.112–1.391	<0.001
>10	1.246	1.076–1.444	0.003
Professional title (ref. = senior nurse or below)			
Supervisor nurse or higher	0.998	0.895–1.113	0.970
Work hours per week (ref. = ≤40)			
>40	1.264	1.175–1.360	<0.001
Night shift in the past 6 months (ref. = no)			
Yes	1.065	0.977–1.161	0.153
Hospital level (ref. = level B major tertiary hospital)			
Level A major tertiary hospital	1.226	1.111–1.352	<0.001
Nurse-to-bed ratio (ref. = 0.36 to <0.40)			
0.40–0.60	0.626	0.503–0.779	<0.001
≥0.60 to 0.79	0.647	0.518–0.808	<0.001

CI = confidence interval; OR = odds ratio; ref. = reference.

## Data Availability

The data in our study are not publicly available because the data contain protected health information, but the research group can provide descriptive data in table form. Requests can be made to Chen Wu or Ke-Fang Wang.
